# APF2: an improved ensemble method for pharmacogenomic variant effect prediction

**DOI:** 10.1038/s41397-024-00338-x

**Published:** 2024-05-27

**Authors:** Yitian Zhou, Sebastian Pirmann, Volker M. Lauschke

**Affiliations:** 1 Department of Physiology and Pharmacology, Karolinska Institutet, Stockholm, Sweden; 2 Center for Molecular Medicine, Karolinska Institutet and University Hospital, Stockholm, Sweden; 3 Computational Oncology Group, Molecular Precision Oncology Program, National Center for Tumor Diseases (NCT) Heidelberg and German Cancer Research Center (DKFZ), Heidelberg, Germany; 4 Helmholtz Information and Data Science School for Health, Karlsruhe/Heidelberg, Germany; 5 Faculty of Biosciences, Heidelberg University, Heidelberg, Germany; 6 Dr Margarete Fischer-Bosch Institute of Clinical Pharmacology, Stuttgart, Germany; 7 University of Tübingen, Tübingen, Germany

**Keywords:** Predictive markers, Genetics research

## Abstract

Lack of efficacy or adverse drug response are common phenomena in pharmacological therapy causing considerable morbidity and mortality. It is estimated that 20–30% of this variability in drug response stems from variations in genes encoding drug targets or factors involved in drug disposition. Leveraging such pharmacogenomic information for the preemptive identification of patients who would benefit from dose adjustments or alternative medications thus constitutes an important frontier of precision medicine. Computational methods can be used to predict the functional effects of variant of unknown significance. However, their performance on pharmacogenomic variant data has been lackluster. To overcome this limitation, we previously developed an ensemble classifier, termed APF, specifically designed for pharmacogenomic variant prediction. Here, we aimed to further improve predictions by leveraging recent key advances in the prediction of protein folding based on deep neural networks. Benchmarking of 28 variant effect predictors on 530 pharmacogenetic missense variants revealed that structural predictions using AlphaMissense were most specific, whereas APF exhibited the most balanced performance. We then developed a new tool, APF2, by optimizing algorithm parametrization of the top performing algorithms for pharmacogenomic variations and aggregating their predictions into a unified ensemble score. Importantly, APF2 provides quantitative variant effect estimates that correlate well with experimental results (R^2^ = 0.91, *p* = 0.003) and predicts the functional impact of pharmacogenomic variants with higher accuracy than previous methods, particularly for clinically relevant variations with actionable pharmacogenomic guidelines. We furthermore demonstrate better performance (92% accuracy) on an independent test set of 146 variants across 61 pharmacogenes not used for model training or validation. Application of APF2 to population-scale sequencing data from over 800,000 individuals revealed drastic ethnogeographic differences with important implications for pharmacotherapy. We thus think that APF2 holds the potential to improve the translation of genetic information into pharmacogenetic recommendations, thereby facilitating the use of Next-Generation Sequencing data for stratified medicine.

## Introduction

Inter-individual variability in drug response that manifests as lack of efficacy or toxicity remains a common concern upon pharmacological therapy. It is estimated that only half of all patients respond to first-line antidepressant therapies [1] and only 4% to 25% of individuals taking the top ten highest-grossing drugs in the United States showed the intended drug response [2]. In addition, adverse drug reaction (ADR) account for around 6.5% of hospital admissions in the UK [3] and up to 12% in Sweden [4]. This number increases to over 15% when patients are older, take different medications at the same time or have comorbidities [5, 6]. Furthermore, ADRs are responsible for 9% of overall healthcare costs [7] and affect approximately one third of novel therapeutics after receiving regulatory approval [8]. It is estimated that 20–30% of such differential drug response stems from genetic factors, i.e., variations in genes that are involved in drug absorption, distribution, metabolism and excretion (ADME), or that encode drug targets [9, 10]. Over the last few decades, a multitude of genetic variants significantly associated with drug response have been identified using forward genetics, particularly for cytochrome P450 (CYP) genes. While these variants are promising pharmacogenomic biomarkers to improve the efficacy and safety of some drugs, increased evidence suggests that they are not sufficient to exhaustively explain the genetically encoded variability in drug response [11–13].

Major developments in sequencing technologies have enabled the profiling of human genomic sequences at the population scale. Whole exome and whole genome sequencing (WES and WGS, respectively) projects have so far identified more than 650 million high-quality variants across over 75,000 human genomes, more than half of which are rare with minor allele frequencies (MAFs) below 0.1% [14]. More than 70,000 variants have been identified in pharmacogenes, of which 80% were novel at the time of analysis and more than 98% were rare with global minor allele frequencies <1% [15, 16]. These rare and novel pharmacogenomic variants are suspected to explain at least part of the missing heritability in drug response [17], however the establishment of their causal effects is intrinsically difficult. For this reason, computational interpretation of rare variations remains to be of great importance in order to consider the full spectrum of pharmacogenomic variation for personalized drug response predictions.

Importantly, most of the available computational algorithms for variant effect predictions are trained on pathogenic variants and consider evolutionary conservation as a key parameter. While these models may perform well for the prioritization of variants implicated in genetic disease, their performance is overall poor when applied to pharmacogenomic variant sets [18, 19]. The reason for this phenomenon is that such algorithms are based on the hypothesis that biologically important regions should be conserved and variants occurring in these regions are thus more likely to exert deleterious effect on the function of the respective gene product. To overcome this limitation, we previously developed an ADME-optimized prediction framework (APF), an ensemble method based on pharmacogenomic reparameterization [20]. The resulting score outperformed previous methods on experimentally characterized variants pharmacogenomic variants, also on genes not included in APF training [21], and allowed quantitative estimates about the magnitude of variant effects on enzyme activity.

A major drawback of conventional prediction algorithms is the limited consideration of structural context due to the lack of high-resolution experimental structures for many pharmacogene products. Recent advances in the prediction of protein folding based on deep learning now allow for the first time to computationally model protein structures to near experimental accuracy with Cα root-mean-square deviation at 95% residue coverage of 0.96 Å [22]. By leveraging these advancements, a novel variant effect predictor termed AlphaMissense was established that integrated information about the structural consequences of missense variations with population frequency inferred labels to predict functional variant [23]. AlphaMissense performed excellently when flagging pathogenic variants; however, its performance on pharmacogenomic data remained to be evaluated.

Here, we leveraged the recent developments in the modeling of protein folding and optimized APF by integrating structural effect predictions. The resulting tool, APF2, predicts the functional impact of pharmacogenomic variants with higher accuracy than APF or AlphaMissense, which is particularly apparent for clinically relevant variations with CPIC guidelines. We furthermore validate APF2 performance on an independent test set of 146 variants with high-confidence functional annotations. By applying APF2 to newly released genomic data from 807,162 individuals, we provide an updated overview of the population-scale variability in pharmacogenes and allowed the refinement of population-specific risk of non-response or adverse drug events for medications with established pharmacogenomic guidelines.

## Methods

### Variant data sources

In total, we generated three non-overlapping variant sets for training, validation and testing of APF2.

Set 1: All missense variants with phenotypic annotations by the Clinical Pharmacogenetics Implementation Consortium (CPIC) were extracted as high evidence set (*n* = 145 variants across 10 genes; Supplementary Table 1). Variants with decreased function or loss-of-function were considered as deleterious, all other variants as functionally neutral.

Set 2: Variants with high-quality experimental characterization data were extracted from the literature. After excluding variants that were already part of Set 1, a total of 385 unique missense variants across 45 pharmacogenes were collected (Supplementary Table 2). Variants with a measured intrinsic clearance <50% and ≥50% of the wild-type enzyme were considered deleterious and functionally neutral, respectively.

Set 3: As test set, we extracted variants from the PharmGKB and ClinVar databases (*n* = 146; Supplementary Table 3). For PharmGKB, variants were annotated as deleterious that impacted drug response or pharmacokinetics with an evidence level of 1 or 2. For ClinVar, we included variants with “drug response” annotation that were reviewed by an expert panel or included in a practice guideline (3 or 4 stars). Variants in pharmacogenes without evidence for an impact on pharmacokinetics or pharmacodynamics were considered functionally neutral.

### Computational variant effect predictions using available algorithms

Variant prediction was performed using a total of 28 computational algorithms. AlphaMissense scores were extracted from https://console.cloud.google.com/storage/browser/dm_alphamissense. Scores for SIFT [24], PolyPhen-2 [25], PROVEAN [26], MutationTaster [27], MutationAssessor [28], VEST [29], ClinPred [30], MutPred [31], APF [20], MetaRNN [32], MetaSVM [33], MetaLR [33], FATHMM [34], FATHMM-MKL [35], CADD [36], DANN [37], REVEL [38], Eigen [39], LRT [40], LIST-S2 [41], DEOGEN2 [42], MVP [43], M-CAP [44], PrimateAI [45], MPC [46], fitCons [47] and GenoCanyon [48] were computed using ANNOVAR [49].

### Definitions

We considered 208 pharmacogenes for consistency with the previous literature [15]. Performance of each of the algorithms was evaluated based on the number of true positives (TP; number of deleterious variants that was correctly identified as deleterious by the respective algorithm), true negatives (TN; number of neutral variants that was correctly identified as neutral by the respective algorithm), false positives (FP; number of neutral variants that was erroneously identified as deleterious by the respective algorithm) and false negatives (FN; number of deleterious variants that was incorrectly identified as neutral by the respective algorithm). The following key metrics were calculated for each algorithm and defined as:

$${{{{{\rm{Sensitivity}}}}}}={TP}/({TP}+{FN})$$

$${{{{{\rm{Specificity}}}}}}={TN}/({TN}+{FP})$$

$${{{{{\rm{Accuracy}}}}}}=({TP}+{TN})/({TP}+{TN}+{FP}+{FN})$$

$${{{{{\rm{Positive}}}}}}\; {{{{{\rm{predictive}}}}}}\; {{{{{\rm{value}}}}}} \, ({{{{{\rm{PPV}}}}}})={TP}/({TP}+{FP})$$

$${{{{{\rm{Negative}}}}}}\; {{{{{\rm{predictive}}}}}}\; {{{{{\rm{value}}}}}} \, ({{{{{\rm{NPV}}}}}})={TN}/({TN}+{FN})$$

$${{{{{\rm{False}}}}}}\; {{{{{\rm{positive}}}}}}\; {{{{{\rm{rate}}}}}} \, ({{{{{\rm{FPR}}}}}})={FP}/({FP}+{TN})$$

$${{{{{\rm{False}}}}}}\; {{{{{\rm{negative}}}}}}\; {{{{{\rm{rate}}}}}} \, ({{{{{\rm{FNR}}}}}})={FN}/({FN}+{TP})$$

Youden’s J was defined on the basis of a receiver operating characteristic (ROC) curve as J = max_x_{Sensitivity + Specificity – 1}. In addition, the area under the ROC curve (AUC) was calculated for each algorithm in R studio (version 2023.09.1).

### Population-scale sequencing data

Genetic variability data for pharmacogenes was extracted from gnomAD v4.0 [50] from 730,947 whole exome and 76,215 whole genome sequences. The data set encompassed information from 37,545 Africans and African Americans, 30,019 Admixed Americans, 14,804 Ashkenazi Jews, 22,448 East Asians, 32,026 Finns, 590,031 non-Finnish Europeans, 45,546 South Asians and 3031 Middle Easterners. The gnomAD dataset was aggregated from different projects and reprocessed using uniform pipelines for increased consistency. The overall population is balanced between males and females. Variant carrier frequency was calculated by aggregating variant frequencies using the Hardy-Weinberg equation. We considered variants with MAFs ≥1% as common and variants with MAFs <1% as rare.

## Results

### Benchmarking pharmacogenomic variant predictions

**Fig. 1:**
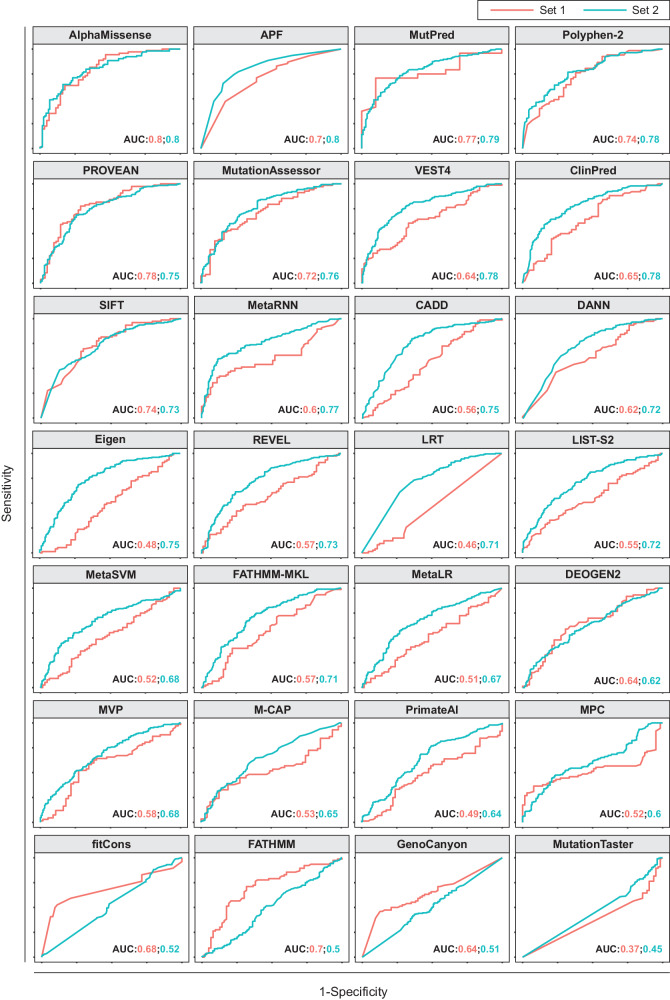
Performance of 28 computational variant effect predictors on different benchmarking sets. Set 1 contained 145 missense variants with high clinical evidence from CPIC while Set 2 refers to 385 non-overlapping missense variants with available in vitro functionality data. Areas under the receiver operating characteristic curves (AUCs) are shown.

To evaluate the performance of available computational effect predictors on pharmacogenomic data, we first benchmarked 28 commonly used algorithms on 145 variants with pharmacogenomic annotations from CPIC (Set 1) and 385 variants with high-quality experimental functionality data (Set 2; see Methods). The best performing methods were AlphaMissense and APF (Fig. 1; Table 1), which achieved the overall highest AUC. Notably, AlphaMissense exhibited excellent specificity (94%) and PPV (85%) while its sensitivity was relatively low (33%). In contrast, APF was more balanced with an overall higher sensitivity (79%) than specificity (66%). MutPred, a method that models a broad range of structural and functional properties, including secondary structure, transmembrane topology and macromolecular binding also performed well; however, 49% of variants could not be predicted. Among the algorithms integrated into APF (MutationAssessor, PROVEAN, VEST, CADD and LRT), the former three were among the top performing methods (AUC ≥ 0.75), whereas predictions of CADD (AUC = 0.7) and LRT (AUC = 0.67) were less accurate when using standard parameters. Combined, these results suggested that incorporation of structural information based on AlphaMissense might improve algorithmic performance, particularly by increasing test specificity.

**Table 1:** Benchmarking 28 algorithms using the combined dataset.

		SEN	SPE	ACC	PPV	NPV	FPR	FNR	J	AUC	Missing
AlphaMissense	≥0.564	0.33	0.94	0.64	0.85	0.59	0.06	0.67	0.28	0.80	177 (33%)
APF	>0.5	0.79	0.66	0.73	0.73	0.73	0.34	0.21	0.45	0.78	6 (1%)
MutPred	>0.5	0.88	0.40	0.68	0.67	0.70	0.60	0.12	0.28	0.78	258 (49%)
PolyPhen-2	>0.446	0.76	0.65	0.71	0.70	0.72	0.35	0.24	0.41	0.76	131 (25%)
PROVEAN	<−2.282	0.81	0.53	0.68	0.66	0.73	0.47	0.19	0.35	0.76	23 (4%)
MutationAssessor	>1.9	0.81	0.54	0.68	0.64	0.74	0.46	0.19	0.35	0.75	114 (22%)
VEST3	>0.9	0.17	0.96	0.54	0.82	0.51	0.04	0.83	0.13	0.75	0 (0%)
ClinPred	≥0.5	0.63	0.72	0.67	0.71	0.64	0.28	0.37	0.35	0.74	15 (3%)
VEST4	>0.5	0.58	0.79	0.68	0.76	0.63	0.21	0.42	0.37	0.74	6 (1%)
SIFT	≤0.05	0.79	0.56	0.68	0.66	0.71	0.44	0.21	0.35	0.74	23 (4%)
MetaRNN	≥0.5	0.59	0.82	0.70	0.78	0.64	0.18	0.41	0.41	0.72	15 (3%)
CADD	>15	0.90	0.41	0.67	0.63	0.78	0.59	0.10	0.30	0.70	6 (1%)
DANN	>0.96	0.85	0.43	0.65	0.63	0.71	0.57	0.15	0.28	0.69	6 (1%)
Eigen	≥0	0.61	0.67	0.64	0.67	0.60	0.33	0.39	0.27	0.69	6 (1%)
REVEL	>0.5	0.41	0.83	0.61	0.73	0.56	0.17	0.59	0.24	0.69	15 (3%)
LRT	<0.001	0.73	0.56	0.65	0.65	0.65	0.44	0.27	0.29	0.67	19 (4%)
LIST-S2	>0.5	0.91	0.26	0.59	0.56	0.72	0.74	0.09	0.16	0.67	53 (10%)
MetaSVM	≥0	0.43	0.80	0.60	0.70	0.56	0.20	0.57	0.22	0.64	15 (3%)
fathmm-MKL	>0.5	0.71	0.52	0.62	0.63	0.61	0.48	0.29	0.23	0.64	6 (1%)
MetaLR	≥0.5	0.41	0.76	0.57	0.65	0.54	0.24	0.59	0.16	0.63	15 (3%)
DEOGEN2	>0.5	0.31	0.83	0.57	0.64	0.54	0.17	0.69	0.13	0.62	76 (14%)
MVP	>0.75	0.54	0.60	0.57	0.61	0.53	0.40	0.46	0.14	0.62	60 (11%)
M-CAP	≥0.025	0.59	0.55	0.58	0.62	0.52	0.45	0.41	0.15	0.60	97 (18%)
PrimateAI	≥0.803	0.03	1.00	0.49	0.90	0.48	0.00	0.97	0.03	0.60	28 (5%)
MPC	>0.6	0.16	0.95	0.54	0.78	0.51	0.05	0.84	0.11	0.58	28 (5%)
fitCons	>0.7	0.95	0.06	0.53	0.53	0.50	0.94	0.05	0.01	0.57	6 (1%)
FATHMM	≤−1.5	0.79	0.34	0.57	0.56	0.60	0.66	0.21	0.13	0.55	23 (4%)
GenoCanyon	>0.999	0.73	0.29	0.52	0.54	0.49	0.71	0.27	0.02	0.53	6 (1%)
MutationTaster	>0.5	0.98	0.02	0.53	0.53	0.50	0.98	0.02	0	0.43	7 (1%)

*SEN* Sensitivity, *SPE* specificity, *ACC* accuracy, *PPV* positive predictive value, *NPV* negative predictive value, *FPR* false positive rate, *FNR* false negative rate, *J* Youden Index, *AUC* area under the receiver operating characteristic curve.

### Developing APF2 by integrating structural information into the established ADME Prediction Framework

**Fig. 2:**
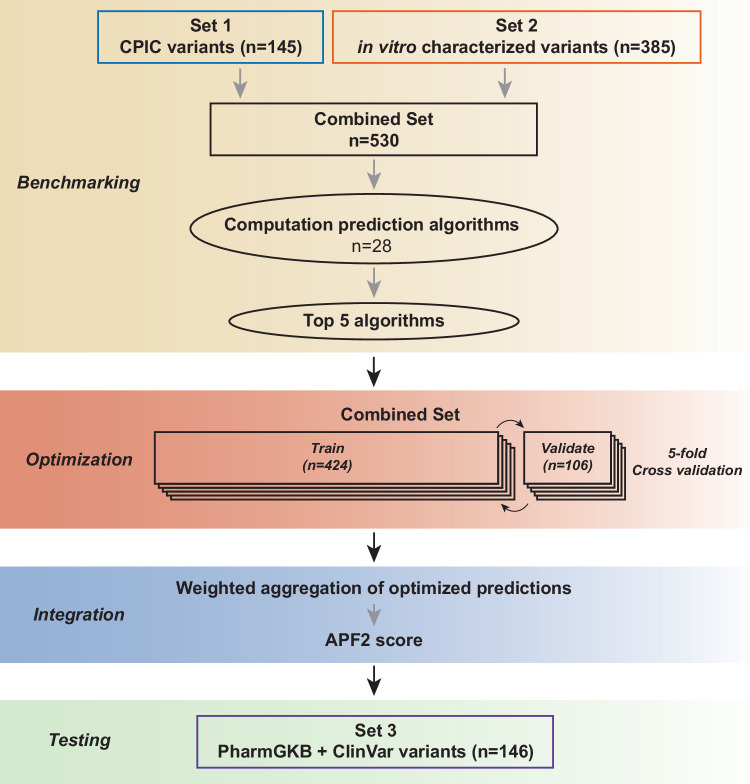
Schematic depiction of the APF2 developmental workflow. Set 1 and Set 2 were integrated and the algorithms exhibiting the best performance (AlphaMissense, PROVEAN, MutationAssessor, PolyPhen-2, VEST) were selected and optimized using 5-fold cross-validation. After parametrization, the optimized algorithms were integrated into the APF2 ensemble score. APF2 performance was finally tested on an independent variant set (Set3, consisting of an additional 146 variants with high confidence annotations) to examine its performance on unseen pharmacogenomic variant data.

The incorporation of structural predictions into a new prediction framework was conducted in two steps - first, we optimized algorithm parametrization for pharmacogenomic variations and second, we aggregated the predictions from optimized algorithms into a unified ensemble score (Fig. 2). Based on the benchmarking results, we selected the five top performing algorithms, i.e. AlphaMissense, PolyPhen-2, PROVEAN, MutationAssessor and VEST, for parameter optimization. MutPred was not considered due to its large number of missing predictions. To leverage the full potential of the benchmarking set, we performed 5-fold cross-validation by randomly partitioning the combined set (combined Set 1 and Set 2 with a total of *n* = 530 variants) into five subsets and iterated the training on four partitions and validation on the remaining partition.

**Fig. 3:**
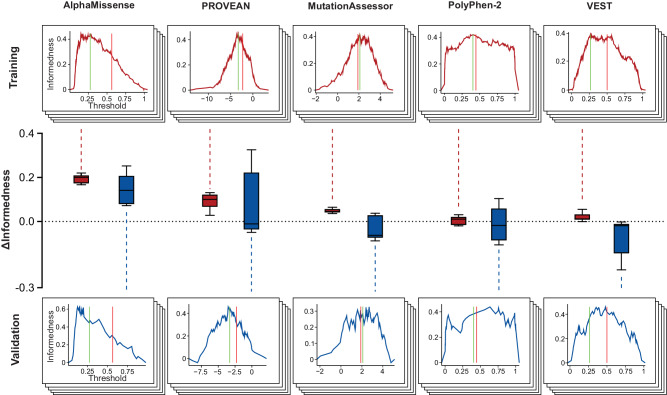
Reparametrization of individual variant effect predictors to optimize performance on pharmacogenomic data. The original parameters of each algorithm (red line) were optimized (green line) to achieve the highest informedness. Following the five-fold cross-validation workflow, training was conducted in 80% of the combined variant sets 1 and 2 (top panels) and validated on the remaining 20% (bottom panels). The changes in informedness (Δinformedness) within training sets and validation sets is illustrated by boxplots in red and blue, respectively.

For threshold optimization, we used Youden’s J that quantifies the likelihood of making an informed decision (see Methods) and selected the value for which J was maximal (Fig. 3). In the training sets, informedness improved considerably for AlphaMissense (ΔJ = 0.19) and PROVEAN (ΔJ = 0.09), whereas only marginal improvements were observed for PolyPhen-2, VEST and MutationAssessor (ΔJ ≤ 0.03). Similar results were obtained when the optimized algorithms were applied to the entire combined set (Table 2). These findings suggest that the optimization approach was robust and improved the predictive accuracy of individual algorithms for pharmacogenomic predictions. We then aggregated the weighted prediction results of the optimized algorithms into the new ensemble score, APF2. The APF2 score can adopt values in the interval [0, 1]. For binary classifications, variants with APF2 scores ≤0.367 were classified as deleterious and variants with a score >0.367 as functionally neutral.

**Table 2:** Parameter optimization of individual algorithms.

	Conventional				Optimized			
	Threshold	SEN	SPE	J	Threshold	SEN	SPE	J
AlphaMissense	≥0.564	0.33	0.94	0.28	≥0.152	0.83	0.63	0.46
PROVEAN	<−2.282	0.81	0.53	0.35	<−3.2925	0.72	0.73	0.44
MutationAssessor	>1.9	0.81	0.54	0.35	>2.08	0.79	0.59	0.38
PolyPhen-2	>0.446	0.76	0.65	0.41	>0.42	0.77	0.65	0.42
VEST4	>0.5	0.58	0.79	0.37	>0.2905	0.75	0.62	0.37

*SEN* Sensitivity, *SPE* specificity, *J* Youden Index.

### Benchmarking of APF2

**Fig. 4:**
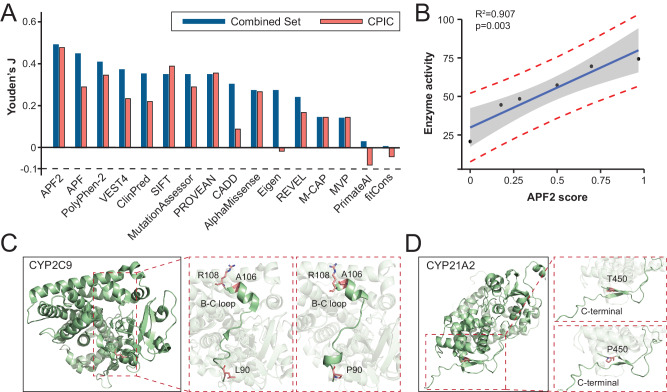
Performance benchmarking of APF2. **A** APF2 outperformed other algorithms, including APF and AlphaMissense, when evaluated on the combined variant set. The improved performance was particularly apparent on clinically important variants with CPIC annotation. **B** APF2 scores significantly correlate with enzyme in vitro activities (R^2^ = 0.907; *p* = 0.003), suggesting that APF2 can predict the quantitative impact of pharmacogenetic variants on gene product function. The confidence interval is shown in gray, the prediction band is indicated by the red dashed lines. **C**, **D** Examples of variants whose functional consequences were incorrectly predicted by previous algorithms that did not consider structural data. The p.L90R variant in CYP2C9 (**C**) and the p.T450P variant in CYP21A2 (**D**) result in pronounced impacts on the tertiary structure of the encoded enzymes, which were correctly predicted as deleterious by AlphaMissense and APF2, but not by other tools.

To test APF2 performance, we first compared classification accuracy to the original APF that does not consider structural information. Notably, APF2 consistently outperformed all individual algorithms (Fig. 4A). The improved accuracy was particularly apparent for clinically relevant variants with CPIC annotations. Trailing APF2 were the original APF, PolyPhen-2 and VEST, whereas algorithms developed on the basis of evolutionary conservation, such as PrimateAI and fitCons, performed very poorly on pharmacogenomic data. Next, we tested the quantitative association between APF2 scores and enzyme function. Importantly, we find that ensemble scores significantly correlate with measured enzyme activity (R^2^ = 0.907, *p* = 0.003; Fig. 4B). These results suggest that APF2 can provide useful information about the magnitude of functional effects of a given pharmacogenetic missense variant.

To demonstrate the molecular basis for the improved performance, we analyzed variations that were correctly identified by APF2, but not by the original APF algorithm that does not consider structural information. CYP2C9*13 (rs72558187; NM_000771.4:c.269T>C) encodes a p.L90P amino acid exchange that abrogates enzyme activity of the respective gene product [51]. Position 90 is upstream of a B-C loop (residues 106–108), which interacts with CYP2C9 substrates, such as S-warfarin. The replacement of leucine by proline distorts this B-C loop and causes side chain turnover of p.A106 and p.R108, thereby reducing substrate interactions and reducing enzyme function (Fig. 4C; and ref. [52]). Another example is the p.T450P amino acid exchange in CYP21A2, which results in a loss of 17-hydroxyprogesterone clearance in vitro [53] and is associated with congenital adrenal hyperplasia due to 21-hydroxylase deficiency [54]. The exchange of a threonine to a proline affects the respective β-sheet structure that is likely required to stabilize the adjacent loop, resulting in destabilization and drastically reduced protein activity (Fig. 4D). Combined, these examples lend support to the importance of structural information for the functional interpretation of pharmacogenetic variations.

Lastly, we tested APF2 on an independent variant set as the gold-standard for evaluating predictive performance. To this end, we obtained pharmacogenomic variants with high level of evidence (*n* = 146) from PharmGKB and ClinVar (Set 3; see Methods). Compared to the commonly used algorithms as well as the best performing tools on the benchmarking set, APF2 was most accurate (92%) with a balanced sensitivity (91%) and specificity (93%) and the highest degree of informedness (J = 0.84). In contrast, the two trailing methods, APF and AlphaMissense, predicted this testing set with a lower accuracy of 85% (Table 3). Overall, these results demonstrated the robustness of APF2 in predicting the functional impact of variations and we thus suggest that it is suitable for application to pharmacogenomic variants with uncertain significance.

**Table 3:** Evaluation of APF2 on PharmGKB and ClinVar variant set.

	SEN	SPE	ACC	PPV	NPV	FPR	FNR	J
APF2	0.91	0.93	0.92	0.95	0.88	0.07	0.09	0.84
VEST4	0.85	0.98	0.91	0.99	0.82	0.02	0.15	0.83
PolyPhen-2	0.94	0.85	0.90	0.91	0.89	0.15	0.06	0.79
AlphaMissense	0.80	0.98	0.87	0.98	0.77	0.02	0.20	0.78
APF	0.94	0.72	0.85	0.82	0.89	0.28	0.06	0.66
MutationAssessor	0.87	0.78	0.84	0.87	0.78	0.22	0.13	0.65
Eigen	0.85	0.81	0.83	0.86	0.79	0.19	0.15	0.66
PROVEAN	0.86	0.75	0.82	0.83	0.80	0.25	0.14	0.62
SIFT	0.84	0.70	0.78	0.80	0.75	0.30	0.16	0.54
CADD	0.95	0.49	0.76	0.72	0.88	0.51	0.05	0.44

*SEN* Sensitivity, *SPE* specificity, *ACC* accuracy, *PPV* positive predictive value, *NPV* negative predictive value, *FPR* false positive rate, *FNR* false negative rate, *J* Youden Index.

### Interrogating functionality of pharmacogenomic variant from population-scale sequencing data

**Fig. 5:**
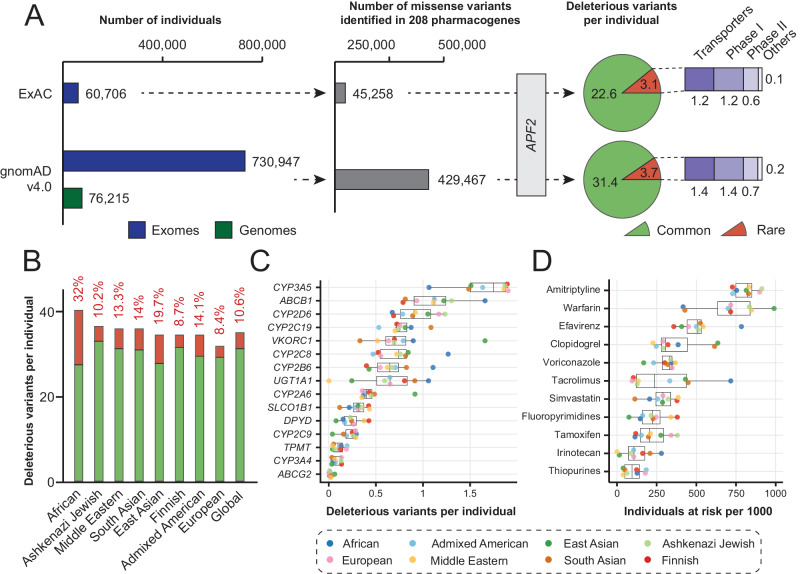
Application of APF2 for pharmacogenomic variant effect predictions at the population-scale. **A** APF2 was utilized to predict the functional impact of pharmacogenomic variants from the legacy ExAC data set and the latest gnomAD release (v4.0). Note that a 13-fold increase in sequenced individuals resulted in a 9.5-fold increase in the number of identified pharmacogenomic variants, whereas estimates for the number of variants per individual were only slightly affected. **B** Stacked column plot showing the number of deleterious variants per individual across eight human populations. The ratio of the genetically encoded functional variability allotted to rare variants (MAF < 1%) is indicated in red. **C** The number of deleterious variants per individual is shown for 15 relevant pharmacogenes. **D** By integrating functional variant data with available CPIC guidelines, the fraction of individuals that would benefit from genetically guided dose adjustments or a change in medication is shown as “individuals at risk/1000 individuals” for different human populations.

Next, we utilized APF2 to evaluate the functional impact of genetic variants across the human pharmacogenome. To this end, we mined variant data of 208 genes from 730,947 whole exome and 76,215 whole genome sequences provided by gnomAD v4.0. Overall, APF2 predicted over 100,000 variants to be deleterious (Supplementary Table 4), resulting in each individual harboring on average a total of 35.1 pharmacogenomic variants, of which 10.6% (3.7) were rare variants with a minor allele frequency (MAF) below 1% (Fig. 5A). Notably, compared to a previous exome data set of 60,706 individuals [55], the aggregated frequencies of deleterious variants increased only modestly at the global scale. When we further stratified the analysis by different human populations, it was revealed that Africans on average carry the highest number of functional pharmacogenomic variants (40.4 per individual), followed by Ashkenazi Jews (36.7 per individual) and Middle Easterners (36 per individual), whereas numbers were more than 20% lower in European individuals (31.8 functional variants; Fig. 5B). Importantly, the functional contribution from rare variant was very high in Africans (32%), possibly due to the large number of rare variants derived from population admixture [56]. These results emphasize the increased variability of the African superpopulation and underscore the importance of genetic profiling with high ethnogeographic resolution.

We then utilized the population-specific variant data to estimate the number of deleterious variants per individual for actionable pharmacogenes (Fig. 5C). Owing to the globally high prevalent splicing defect variant CYP3A5*3, all populations harbored on average at least one deleterious *CYP3A5* variant per individual. Similarly, more than one deleterious *ABCB1* variant per individual was observed in Africans, Ashkenazi Jews, East Asians, Admixed Americans and Middle Easterners, primarily due to the synonymous variant at rs1045642 (NP_000918.2:p.Ile1145=; sometimes referred to as C3435T) for which there is some evidence of functional importance [57, 58]. In contrast, the ABC transporter BCRP, encoded by *ABCG2*, is >50-fold less variable. We then leveraged these genetic variation data to infer the fraction of individuals at risk based on established CPIC recommendations (Fig. 5D). Individuals at risk in this context was defined as the fraction of each ethnogeographic group who would be expected to benefit from dose adjustments or alternative medications based on their genotype. Over 70% of individuals across populations are estimated to exhibit poor response or avoidable adverse events upon treatment with standard doses of amitriptyline, aligning with clinical trial data [59]. Similarly, a large number of patients are predicted to benefit from dose adjustments of warfarin; however, the fraction of at-risk individuals differs substantially between populations from 40% in Africans and South Asians to 99% in East Asians. These findings match the 59–75% reduced doses prescribed in East Asia compared to the rest of the world (2.45 mg daily vs. 4–8.76 mg daily) [60]. For clopidogrel, our results predict that on average 30% of individuals of European ancestry taking clopidogrel would benefit from dose adjustments or alternative antiplatelet treatment, which again matches well with previous clinical findings that showed that up to 37% of patients do not respond appropriately to clopidogrel [61]. The predicted numbers of individuals at risk can differ substantially between populations. For instance, for irinotecan, the fraction differed between 0.1/1000 individuals in the Middle East compared to 280/1000 for Africans. Similarly, relative risk differed >7-fold for tacrolimus where 95/1000 European individuals would benefit from genetically guided prescriptions compared to 717/1000 Africans. These results indicate the importance of considering ethnogeographic information for drug response and the personalization of treatment.

## Discussion

Over the last decades, a multitude of computational algorithms have been developed to predict the functional consequences of missense variants. These either leverage statistical methods based on sequence information or adopt machine learning on existing data to infer variant effect. Evolutionary conservation has been the predominant feature for either of these approaches. However, pharmacogenomic variations are often not highly conserved, which reduces the predictive power of conservation-based tools [62]. Moreover, as many algorithms consider variants that are very common in the general population as functionally neutral, there are multiple functionally important variations, such as the variants defining haplotypes CYP2C8*2 (MAF = 15.2% in Africans), CYP2C19*2 (MAF > 26% in Asian populations), CYP2D6*4 (MAF = 19.6% in Europeans) and CYP2D6*10 (MAF = 57.3% in East Asians) that are incorrectly assigned during training, reducing algorithm performance and accuracy [63]. As a consequence, most tools are designed to predict pathogenic rather than deleterious variations.

To overcome this issue, both gene-specific [64–66] and general purpose pharmacogenomic algorithms [20, 67] have been developed. However, these tools are based on supervised learning and critically rely on deleterious and neutral variant sets with accurate labels with no or only limited consideration of structural information. Here, we extended these approaches by integrating AlphaMissense scores, an approach that they did not use labeled pathogenic variants as training. Indeed, the resulting APF2 score improved predictive performance on an unseen variant set that also included variations in genes that were not part of model training. As such, high accuracy is achieved while avoiding model circularity, which constitutes a common issue known to result in inflated performance estimates [68]. The use of an ensemble method is moreover in line with current guidelines by the American College of Medical Genetics (ACMG), which recommends using a concordance-based approach that integrates predictive results from several prediction algorithms [69].

Notably, high-quality functional data from in vitro experiments are required for the training and validation of variant effect predictors. For APF2, we utilized a total of 530 variants distributed across 45 pharmacogenes for model development, including genes encoding CYPs and other phase I enzymes, phase II enzymes as well as drug transporters. While this is an increase of almost 60% compared to the original APF, the extent of pharmacogenomic training data remains limited. Notably, recent advances in deep mutational scanning drastically increase the numbers of variants with functional annotations. For instance, activity data has been generated for >6000 variants in *CYP2C9* [70] as well as for >2900 in *NUDT15* [71]. However, we here decided to omit these data to avoid skewing the data towards one or few genes. With the accumulation of such data sets, we anticipate that these can provide powerful resources for the further refinement of pharmacogenomic prediction algorithms.

Analysis of genome-scale sequencing data from a total of 807,162 individuals (gnomAD v4.0) revealed a total of 31.4 common and 3.7 rare pharmacogenetic variants with putative functional consequences. These numbers are similar to previous estimates based on 60,706 exomes from the legacy ExAC project derived by APF [15] or APF2, suggesting that a further increase in cohort sizes has, if at all, only minor impacts on the overall number of aggregated pharmacogenetic variations per individual. The current analyses also confirm pronounced population differences. Consequently, although estimates for the overall aggregated frequency of pharmacogenetic variants might reach saturation at the global scale, there is clearly a need for more refined genetic signatures with high ethnogeographic resolution.

While APF2 outperformed previous algorithms, multiple limitations remain. Firstly, APF2 can only predict quantitative variant effects for reduced function of the gene product, whereas gain-of-function effects cannot be predicted by the current version. Secondly, APF2, like all other algorithms, cannot identify substrate-specific effects. For instance, CYP2C8*3 is thought to increase clearance of glitazones, whereas the same allele is associated with reduced metabolism of ibuprofen and paclitaxel [72]. APF2 only provides a single score and, in the case of CYP2C8*3, identifies the underlying variants as deleterious, thus not hinting at potential differences between substrates. Furthermore, APF2 only evaluates single variants and is currently not able to provide interpretations of combinatorial variant effects. This limitation might be particularly pronounced for *CYP2A6* and *CYP2D6* genes where complex haplotype structures are routinely identified. Lastly, the tool is limited to missense variants, whereas regulatory variants or variations that result in splicing defects or ribosomal stalling cannot be detected.

Translating genomic sequence data into treatment recommendations faces the important challenge of how to consider variants for which no epidemiological or experimental functionality data is available. Currently, the application of computational variant interpretation is limited to research settings. In this context, it has been suggested that, when sequencing data are available, predicting the function of variants of unknown significance can act as one piece of evidence to assist clinical decision making, for instance by flagging carriers of putatively deleterious variants for high-intensity surveillance or therapeutic drug monitoring [73]. Indeed, application of APF2 to population-scale pharmacogenomic data identified that up to 90% patients of European and Ashkenazi Jewish ancestry would benefit from dose adjustments when undergoing amitriptyline therapy and, overall, 78% of African patients are at risk of poor response or adverse events when treated with efavirenz. However, stringent trials with high ethnogeographic resolution are needed to evaluate whether sequencing coupled with computational evaluation of unknown variants can improve personalized response predictions in well-defined therapeutic contexts and whether those interventions constitute a cost-effective allocation of health care resources.

In summary, we have extended the established APF algorithm by integrating structural information inferred by AlphaMissense. The resulting tool, APF2, predicts the functional impact of pharmacogenomic variants with higher accuracy, particularly for clinically relevant variations with CPIC guidelines. We furthermore demonstrate 92% accuracy with balanced sensitivity and specificity on an independent test set of 146 variants from PharmGKB and ClinVar. Combined, these findings suggest that integration of structural data provides a further step towards reliable pharmacogenetic variant effect prediction, which might facilitate the translation of personal sequencing data into personalized pharmacogenetic advice.

### Supplementary information

Supplementary Table 1

Supplementary Table 2

Supplementary Table 3

Supplementary Table 4

## Data Availability

Aggregated variant data is available via gnomAD (https://gnomad.broadinstitute.org/), AlphaMissense scores can be accessed at https://console.cloud.google.com/storage/browser/dm_alphamissense. Both repositories are publicly available. All relevant information from variants that were used for model training, validation and testing are provided in Supplementary Tables S1–S3.
